# Application of a delta-6 desaturase with α-linolenic acid preference on eicosapentaenoic acid production in *Mortierella alpina*

**DOI:** 10.1186/s12934-016-0516-5

**Published:** 2016-06-30

**Authors:** Haisu Shi, Haiqin Chen, Zhennan Gu, Hao Zhang, Wei Chen, Yong Q. Chen

**Affiliations:** State Key Laboratory of Food Science and Technology, School of Food Science and Technology, Jiangnan University, 1800 Lihu Avenue, Wuxi, 214122 Jiangsu People’s Republic of China; Synergistic Innovation Center for Food Safety and Nutrition, Wuxi, 214122 People’s Republic of China; Departments of Cancer Biology and Biochemistry, Wake Forest School of Medicine, Winston-Salem, NC 27157 USA

**Keywords:** Delta 6 desaturase, *Mortierella alpina*, Eicosapentaenoic acid, Peony seed oil, Peony seed meal

## Abstract

**Background:**

Delta-6 desaturase (FADS6) is a key bifunctional enzyme desaturating linoleic acid (LA) or α-linolenic acid (ALA) in the biosynthesis of polyunsaturated fatty acids (PUFAs). In previous work, we analyzed the substrate specificity of two FADS6 enzymes from *Mortierella alpina* ATCC 32222 (MaFADS6) and *Micromonas pusilla* CCMP1545 (MpFADS6), which showed preference for LA and ALA, respectively. We also clarified the PUFA profiles in *M. alpina*, where these lipids were synthesized mainly via the ω6 pathway and rarely via the ω3 pathway and as a result contained low ALA and eicosapentaenoic acid (EPA) levels.

**Result:**

To enhance EPA production in *M. alpina* by favoring the ω3 pathway, a plasmid harboring the MpFADS6 gene was constructed and overexpressed in a uracil-auxotrophic strain of *M. alpina* using the *Agrobacterium tumefaciens*-mediated transformation (ATMT) method. Our results revealed that the EPA production reached 80.0 ± 15.0 and 90.4 ± 9.7 mg/L in MpFADS6 transformants grown at 28 and at 12 °C, respectively. To raise the level of ALA, free form fatty acid was used as exogenous substrate, which increased the EPA production up to 114.5 ± 12.4 mg/L. To reduce the cost of EPA production in *M. alpina*, peony seed oil (PSO) and peony seed meal (PSM) were used as source of ALA, and EPA production was improved to 149.3 ± 7.8 and 515.29 ± 32.66 mg/L by supplementing with 0.1 % PSO and 50 g/L PSM, respectively. The EPA yield was further increased to 588.5 ± 29.6 mg/L in a 5-L bioreactor, which resulted in a 26.2-fold increase compared to EPA production in wild-type *M. alpina*. In this work, we have significantly enhanced EPA production through overexpression of a FADS6 desaturase with preference for ALA, combined with supplementation of its substrate.

**Conclusion:**

An ALA-preferring FADS6 from *M. pusilla* CCMP1545 was applied to enhance EPA production in *M. alpina*. By exogenous addition of peony seed oil or peony seed meal, EPA production was further increased in flasks and fermenters. This research also highlights the value of peony seed meal which can be converted to a high value-added product containing EPA, and as a way to increase the EPA/AA ratio in *M. alpina*.

**Electronic supplementary material:**

The online version of this article (doi:10.1186/s12934-016-0516-5) contains supplementary material, which is available to authorized users.

## Background

Polyunsaturated fatty acids (PUFAs) are recognized as essential nutrients, and in particular the dietary intake of ω3-PUFAs such as eicosapentaenoic acid (EPA, 20:5^Δ5, 8, 11, 14, 17^) plays crucial roles in infant growth and adult health [1]. Many studies have indicated that EPA decreases the risk of many diseases. Currently, marine fish is the main source of EPA, but the depletion of global fisheries and the pollution of the marine environment [2] have prompted the search for alternative sources to meet our necessary intake of EPA. Oleaginous microorganisms such as *Mortierella alpina* (*M. alpina*) [3], *Pythium irregulare* (*P. irregulare*) [4], *Micromonas pusilla* (*M. pusilla*) [5], *Phaeodactylum tricornutum* (*P. tricornutum*) [6] and *Yarrowia lipolytica* (*Y. lipolytica*) [7] are innovative sources of EPA. It is notable that *P. irregulare*, *M. pusilla* and *P. tricornutum* produce lower levels of PUFA, and that the desaturase/elongase system of wild-type *Y. lipolytica* is incomplete.

By contrast, the *Mortierella* genus presents several key advantages for PUFA production: its members are highly oleaginous strains and have previously been extensively studied for the practical production of arachidonic acid (AA, 20:4^Δ5, 8, 11, 14^) [8]; their PUFAs were found to be safe for human consumption; and their desaturase/elongase systems are intact. In particular, *M. alpina* ATCC 32222 (*M. alpina*), whose genome has previously been characterized in our laboratory [9], has ability to synthesize PUFAs and accumulate them up to about 40–50 % of its dry cell weight (DCW). But EPA production in *M. alpina* is limited. PUFA production in *M. alpina* begins with a delta-6 desaturation (since the delta 8 pathway does not exist in this organism) as the first committed steps of two separate pathways that lead to ω6- and ω3- PUFA synthesis (Additional file 1: Fig. S1) [10]. Previously, delta 6 desaturase in *M. alpina* (MaFADS6-I) was confirmed to be better able to convert linoleic acid (LA, 18:2^Δ9,12^) to γ-linolenic acid (GLA, 18:3^Δ6,9,12^) than to convert α-linolenic acid (ALA, 18:3^Δ9,12,15^) to stearidonic acid (SDA, 18:4^Δ6,9,12,15^) [11]. This revealed that the ω6 pathway of conversion of LA to AA is the main route for PUFA production in *M. alpina*, with only minor desaturation or elongation being achieved in the ω3 pathway. Thus, we reasoned that transformation of an exogenous ALA-preferring FADS6 gene in *M. alpina* could enhance ω3-PUFA accumulation in the ω3 pathway, in parallel with its main pathway (Additional file 1: Fig. S1). This could result in the production of both ω6- and ω3-PUFAs, potentially including a few desirable products such as EPA. Previously, the catalytic efficiency of FADS6 from various species with LA and ALA substrates was summarized [11]; *M. pusilla*, a type of marine microalgae whose FADS6 (MpFADS6) appears to function as an acyl-CoA desaturase, was found to have the highest preference for the ω3 substrate (ALA) compared to other plants and fungi. The conversion efficiency of MpFADS6 for LA and ALA was found to be 4.9 and 63.0 % respectively [5]. MpFADS6 presented the highest ALA/LA conversion ratio by far. Accordingly, we selected the MpFADS6 gene as the target gene transformed into *M. alpina* in our present study.

The objectives of this research were to integrate the MpFADS6 gene into the *M. alpina* genome, to investigate the potential for recombinant fungal production of EPA, and to determine the feasibility of using tree peony seed oil (PSO) and its processing byproduct, peony seed meal (PSM) as substrates for the fermentation process. Little work has been performed utilizing PSO processing wastes and byproducts as substrates for fungal oil production, although other food wastes and byproducts have been widely used. O’Brien et al. [12] determined that sweet whey permeate (lactose) was an excellent substrate for the production of a high-EPA-content lipid by the filamentous fungus *P.irregulare*. Cheng et al. [13] investigated the potential production of EPA and AA using crude soybean oil (SBO), a sucrose waste stream (SWS) and a soymeal waste stream (SMW) as substrates for the fungal fermentation process. These studies encouraged the use of food processing wastes and byproducts as potential substrates for fungal oil production.

In this paper, we first cloned both MaFADS6-I and MpFADS6 genes, constructed binary plasmids pBIG2-ura5s-MaFADS6-I and pBIG2-ura5s-MpFADS6, and then use the *Agrobacterium tumefaciens* mediated transformation (ATMT) method to overexpress each FADS6 separately in *M. alpina* uracil-auxotrophic strains. Second, transgenic fungi were grown in shaken flasks to determine whether they presented increased EPA production under constant temperature and low temperature conditions. Third, the substrates including the free form of fatty acid, PSO and PSM were separately added in transgenic fungi; and the optimal amount of PSO or PSM was selected for EPA production. Finally, EPA yield was further investigated in a 5-L fermenter at 50 g/L PSM supplementation. To our knowledge, this is the first report where EPA production in oleaginous fungus was increased by introduction of a heterologous FADS6 gene and addition of PSO or PSM as substrate.

## Results

### Overexpression of MpFADS6 gene in *M. alpina*

To construct the binary plasmids, a 1374-bp fragment was amplified using pBIG-FMa/pBIG-RMa primers, and a 1392-bp fragment was amplified using pBIG-FMp/pBIG-RMp primers (Additional file 2: Fig. S2a, b). Both fragments were ligated into pBIG2-ura5s-ITs vector, constructing pBIG2-ura5s-MaFADS6-I and pBIG2-ura5s-MpFADS6 vectors (Additional file 2: Fig. S2c, d). The resulting pBIG2-ura5s-MaFADS6-I and pBIG2-ura5s-MpFADS6 were electrotransformed separately into *A. tumefaciens.* After co-cultivation of *M. alpina* uracil-auxotrophic strain and each positive *A. tumefaciens* transformant, ten putative transformants integrating the MpFADS6 gene and eight putative transformants integrating the MaFADS6-I gene appeared in SC-CS medium. After subculturing for three generations on GY plates, all 18 transformant spores identified were inoculated in GY medium.

PCR amplification was used to identify MpFADS6 and MaFADS6-I gene fragments in genomic DNA from the transformants. We obtained two positive strains among eight putative MaFADS6 transformants, presenting a 1374-bp fragment with FMa/RMa primers and designated Ma-MaFADS6-1 and Ma-MaFADS6-2, and three positive strains among ten putative MpFADS6 transformants, presenting a 1392-bp fragment with FMp/RMp primers and designated Ma-MpFADS6-1, Ma-MpFADS6-2 and Ma-MpFADS6-3 (Additional file 2: Fig. S2e, f). Also, the presence of both ura5 and MaFADS6-I/MpFADS6 expression units in the genomic DNA was confirmed by PCR using promoter- and terminator-specific primers (HisproF/TrpCR). Our results showed that a 1553-bp (MaFADS6-I expression unit) or 1571-bp (MpFADS6 expression unit) fragment along with an 818-bp fragment (ura5 expression unit) was amplified while an 818-bp fragment (ura5 expression unit) along with a 352-bp fragment (ITs expression unit) was amplified in pBIG2-ura5s-ITs plasmid (negative control) using HisproF/TrpCR primers (Additional file 2: Fig. S2g, h).

**Fig. 1:**
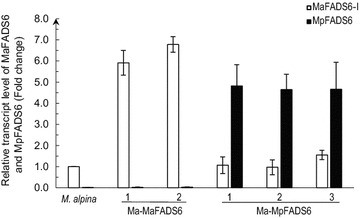
Relative transcript level of MaFADS6-I and MpFADS6 in Ma-MaFADS6-I, Ma-MpFADS6 transformants and the control strain (wild-type *M. alpina*). The *open bars* represent the MaFADS6-I transcript level and the *black bars* represent the MpFADS6 transcript level. All values are the means of three independent experiments and *error bars* represent standard deviations. Relative transcript level of the control strain was defined as 1

The expression levels of the MaFADS6-I and MpFADS6 genes in all samples (including WT *M. alpina*, Ma-MaFADS6-1/2 transformants and Ma-MpFADS6-1/2/3 transformants) were analyzed after 7-day cultivation. RT-qPCR was performed to determine their transcript level. The results revealed that the transcript level of the MaFADS6-I gene in Ma-MaFADS6-1/2 transformants was approximately sixfold higher than that in WT *M. alpina*, and the transcript level of the MpFADS6 gene in each Ma-MpFADS6 transformant was equivalent to that of the MaFADS6-I gene in each Ma-MaFADS6-I transformant after correction for the endogenous MaFADS6-I gene transcript level (Fig. 1).

### Fatty acid profile of transformants without substrate supplementation

To eliminate the effect of endogenous MaFADS6-I when analyzing the changes of fatty acid profiles between WT *M. alpina* and Ma-MpFADS6 transformants, transformation of MaFADS6-I to *M. alpina* uracil-auxotrophic strain was used as a negative control.

**Fig. 2:**
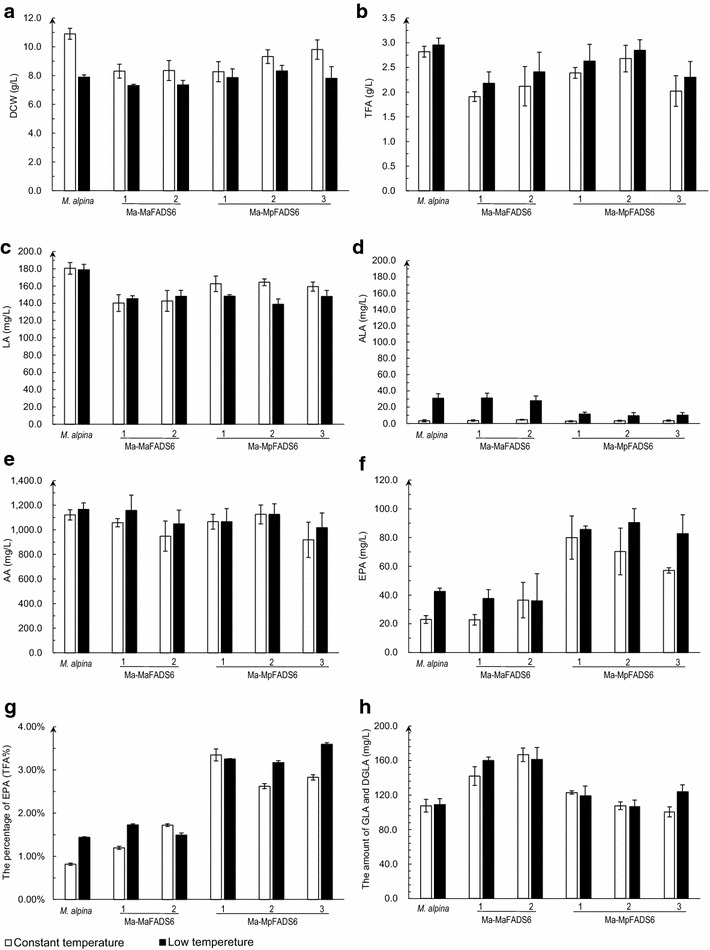
The effect of constant temperature and low temperature conditions on each strain growth and principal fatty acid profiles without any substrate. DCW (dry cell weight) (**a**), TFA (total fatty acids) (**b**), LA level (**c**), ALA level (**d**), AA level (**e**), EPA level (**f**), the percentage of EPA (**g**) and the amount of GLA and DGLA (**h**) were determined in the control strain (WT *M. alpina*) and transformants (including Ma-MaFADS6-I-1, Ma-MaFADS6-I-2, Ma-MpFADS6-1, Ma-MpFADS6-2 and Ma-MpFADS6-3). Mycelia were cultured under constant temperature (7 days at 28 °C) or low temperature (3 days at 28 °C and then at 12 °C for 8 days) in Broth medium. All values are the means of three independent experiments and error bars represent standard deviations. TFA (g/L) = M_TFA_ of 50 mg freeze-dried cells × M_CDW_ of 50 mL cultures/50 mg × 20

All transformants and WT *M. alpina* were grown in Broth medium at 28 °C (constant temperature) for 7 days, and their fatty acid profiles were determined. No significant differences were found in dry cell weight, total fatty acids, LA, ALA and AA levels among these transformants (Fig. 2a–e), whereas the EPA level in Ma-MpFADS6 transformants was increased up to 80.0 ± 15.0 mg/L compared to 22.99 ± 2.7 mg/L in WT *M. alpina* (Fig. 2f). The percentage of EPA (as % of TFA) accounted for 3.35 ± 0.14 % in Ma-MpFADS6-1 transformants compared to 0.82 ± 0.02 % in WT *M. alpina* (Fig. 2g). In negative control MaFADS6-I, the contents of the amount of GLA and DGLA (Dihomo-γ-linolenic acid, 20:3^Δ8,11,14^) were about 40 mg/L higher than that in *M. alpina* (Fig. 2h). Low amounts of extracellular fatty acids were detected in the supernatant of all cultures.

Our results revealed that the over-expression of MpFADS6 in WT *M. alpina* can improve the metabolic flux of ALA to the synthesis of EPA, but endogenous ALA level was too low to be an effective substrate for MpFADS6 (Fig. 2d). To increase the endogenous ALA level, the conversion efficiency of LA to ALA has to be enhanced. The FADS15 enzyme presents a higher conversion efficiency at low temperature [14]. Therefore, mycelia were cultured for 3 days at 28 °C and then for 8 days at 12 °C (low temperature) in Broth medium. Our result showed that the DCW of all strains decreased slightly compared to the constant temperature experiment, whereas TFA levels presented a slight increase (Fig. 2a, b). No significant change was found in LA and AA levels between the constant temperature and low temperature conditions (Fig. 2c, e). EPA production reached 90.4 ± 9.7 mg/L, which is only 10.4 mg/L more than under constant temperature (Fig. 2f). The ALA level in WT *M. alpina* was still low using the low temperature protocol, although it increased from 3.3 ± 1.1 to 31.0 ± 5.6 mg/L, whereas ALA levels in transformants were lower than those in WT *M. alpina* (Fig. 2d).

### Fatty acid profile with addition of exogenous ALA

Although low temperature can prompt the conversion of LA to ALA in *M. alpina*, the activity of MaFADS15 was still too low to produce adequate amount of ALA, and the EPA production was only marginally improved. We attempted to solve this problem by supplementing cultures with exogenous free fatty acid in cultures to supply adequate ALA and enhance EPA production. Considering the cost of free fatty acid, a low level [0.1 % (m/v)] was added in cultures.

**Fig. 3:**
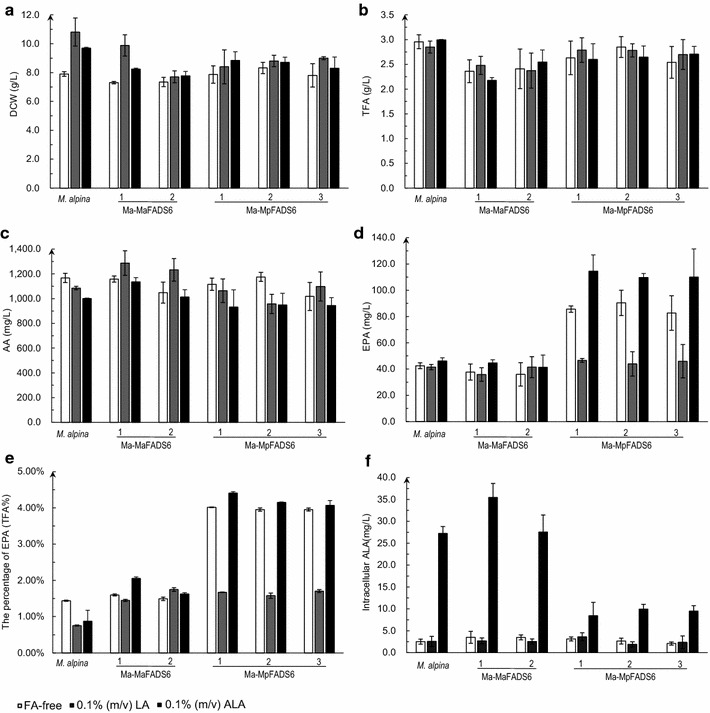
The effect of free form of fatty acid on each strain growth and principal fatty acid profiles. DCW (**a**), TFA (**b**), AA level (**c**), EPA level (**d**), the percentage of EPA (**e**), intracellular ALA level (**f**) were determined in the control strain (WT *M. alpina*) and transformants (including Ma-MaFADS6-I-1, Ma-MaFADS6-I-2, Ma-MpFADS6-1, Ma-MpFADS6-2 and Ma-MpFADS6-3). Mycelia were cultured for 3 days at 28 °C and then at 12 °C for 8 days in Broth medium containing FA-free, 0.1 % (m/v) LA or 0.1 % (m/v) ALA as the exogenous substrate. All values are the means of three independent experiments and *error bars* represent standard deviations. TFA (g/L) = M_TFA_ of 50 mg freeze-dried cells × M_CDW_ of 50 mL cultures/50 mg × 20

No significant changes were observed in DCW and TFA among all strains after addition of exogenous free fatty acid (Fig. 3a, b). Addition of exogenous LA resulted in a slight increase of AA in Ma-MaFADS6 transformants compared to WT *M. alpina* (Fig. 3c), but the level of EPA in Ma-MpFADS6 transformants was noticeably decreased, approximately 40 mg/L less than that in unsupplemented conditions (Fig. 3d). After addition of exogenous ALA, all of the Ma-MpFADS6 transformants presented a conspicuous increase in the level of EPA, which reached 4.17 ± 0.10 % of the TFA in Ma-MpFADS6-1 transformant; this represents a 2.5-fold increase relative to WT *M. alpina*, from 46.2 ± 2.5 to 114.5 ± 12.4 mg/L (Fig. 3d, e). At the same time, the level of intracellular ALA was much lower in Ma-MpFADS6 transformants than in WT *M. alpina* (Fig. 3f).

### Enhancement of EPA production by peony seed oil supplementation

**Fig. 4:**
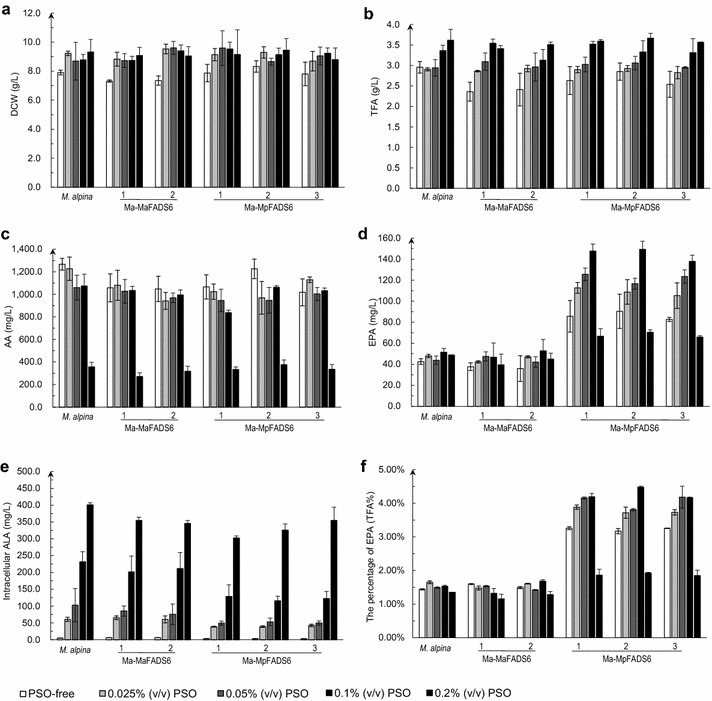
The effect of different concentrations of PSO on each strain growth and principal fatty acid profiles at low temperature. DCW (**a**), TFA (**b**), AA level (**c**), EPA level (**d**), intracellular ALA level (**e**), the percentage of EPA (**f**) were determined in the control strain (WT *M. alpina*) and transformants (including Ma-MaFADS6-I-1, Ma-MaFADS6-I-2, Ma-MpFADS6-1, Ma-MpFADS6-2 and Ma-MpFADS6-3). Mycelia were cultured for 3 days at 28 °C and then at 12 °C for 8 days in Broth medium containing PSO-free, lower concentrations of PSO [0.025, 0.05 or 0.1 % (v/v) PSO] or higher concentration of PSO [0.2 % (v/v) PSO] as the exogenous substrate. All values are the means of three independent experiments and *error bars* represent standard deviations. TFA (g/L) = M_TFA_ of 50 mg freeze-dried cells × M_CDW_ of 50 mL cultures/50 mg × 20

The cost of free fatty acid as exogenous substrate in culture is too high to justify their use in EPA production in *M. alpina*. To reduce costs, triglycerides can be used as a substrate for EPA production in Ma-MpFADS6 transformants. Peony seed oil (PSO) was selected as a suitable exogenous substrate, because it contains 46 % ALA and 26 % LA (Additional file 3: Fig. S3). Our results revealed that the DCW and TFA of all strains were not significantly altered in cultures after PSO addition (Fig. 4a, b). When PSO concentration was increased, the level of AA was not significantly raised, but it was sharply reduced when the PSO concentration reached 0.2 % (Fig. 4c). Similar results were obtained after supplementing cultures with 0.4 and 0.6 % PSO, data not shown. The Ma-MpFADS6 transformants presented a noticeably higher EPA production with increasing PSO concentrations, with a maximum level of 149.3 ± 7.8 mg/L with 0.1 % PSO, which represents a 3.5-fold increase relative to 42.5 ± 2.7 mg/L in WT *M. alpina* (Fig. 4d). After supplementing cultures with 0.025, 0.05 and 0.1 % PSO in all Ma-MpFADS6 transformants, about half of the intracellular ALA was converted to EPA by MpFADS6, followed by further fatty acid elongation and desaturation steps (Fig. 4e). However, after supplementation with 0.2 % PSO, the level of intracellular ALA in Ma-MpFADS6 transformants was approximately equivalent among all strains (Fig. 4e), and the level of EPA was reduced to half that in the 0.1 % PSO culture (Fig. 4d), which suggests that ALA was not converted to EPA. In Ma-MpFADS6 transformants supplemented with increasing PSO concentrations, the remaining intracellular ALA expressed as a percentage of the intracellular ALA level in WT *M. alpina* showed a growth trend, and from the result of intracellular ALA level in WT *M. alpina*, the percentage of ALA (account for the amount of intra- and extracellular ALA) in cells was slightly decreased with PSO supplementation (58.30, 50.23, 51.40 and 51.60 %, calculated from the data shown in Fig. 4e; Additional file 4: Fig. S4a). The level of extracellular ALA in all of the Ma-MpFADS6 transformants was slightly lower than in WT *M. alpina* (Additional file 4: Fig. S4a). The maximal percentage of EPA (%TFA) reached 4.48 ± 0.03 % with supplementation of 0.1 % PSO (Fig. 4f). The above results confirm that 0.1 % PSO was the optimal concentration for supplementation. Under these conditions, the level of EPA in Ma-MpFADS6 transformants was more than 7.5-fold increased relative to *M. alpina*.

### Further enhancement of EPA production with peony seed meal

**Fig. 5:**
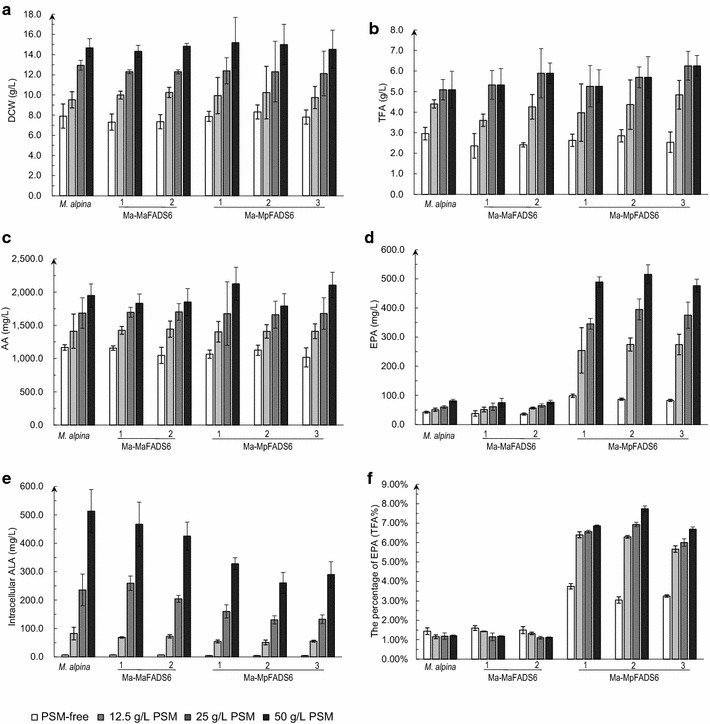
The effect of different concentrations of PSM on each strain growth and principal fatty acid profiles at low temperature. DCW (**a**), TFA (**b**), AA level (**c**), EPA level (**d**), intracellular ALA level (**e**), the percentage of EPA (**f**) were determined in the control strain (WT *M. alpina*) and transformants (including Ma-MaFADS6-I-1, Ma-MaFADS6-I-2, Ma-MpFADS6-1, Ma-MpFADS6-2 and Ma-MpFADS6-3). Mycelia were cultured for 3 days at 28 °C and then at 12 °C for 8 days in Broth medium containing PSM-free, 12.5 g/L PSM, 25 g/L PSM or 50 g/L PSM as the exogenous substrate. All values are the means of three independent experiments and *error bars* represent standard deviations. TFA (g/L) = M_TFA_ of 50 mg freeze-dried cells × M_CDW_ of 50 mL cultures/50 mg × 20

To further reduce costs for EPA production in *M. alpina*, peony seed meal (PSM) was supplemented in cultures. PSM is a rich source of carbon and nitrogen nutrients for microbial growth and its ALA level account for approximately 2 % of its total mass (Additional file 3: Fig. S3). Therefore, PSM can be used as nutrient for transformant growth and also as an exogenous ALA substrate source. Our results showed that the DCW of WT *M. alpina* and all the transformants was significantly increased by supplementing with 12.5, 25 and 50 g/L PSM, up to twofold (with 50 g/L PSM) higher than those of the PSM-free control (Fig. 5a); the levels of TFA and AA were also correspondingly increased (Fig. 5b, c). EPA yield was increased to 274.4 ± 22.4, 394.9 ± 35.6 and 515.29 ± 32.6 mg/L by supplementing with 12.5, 25 and 50 g/L PSM, respectively (Fig. 5d). In addition, as a whole, the level of intra- and extracellular ALA in Ma-MpFADS6 transformants presented similar patterns compared to PSO-supplemented cultures (Fig. 5e; Additional file 4: Fig. S4b). Ma-MpFADS6-2 transformants accumulated EPA to a level of 7.74 ± 0.15 % of the TFA when supplemented with 50 g/L PSM (Fig. 5f).

### Optimization of the EPA yield in a 5-L fermenter

**Fig. 6:**
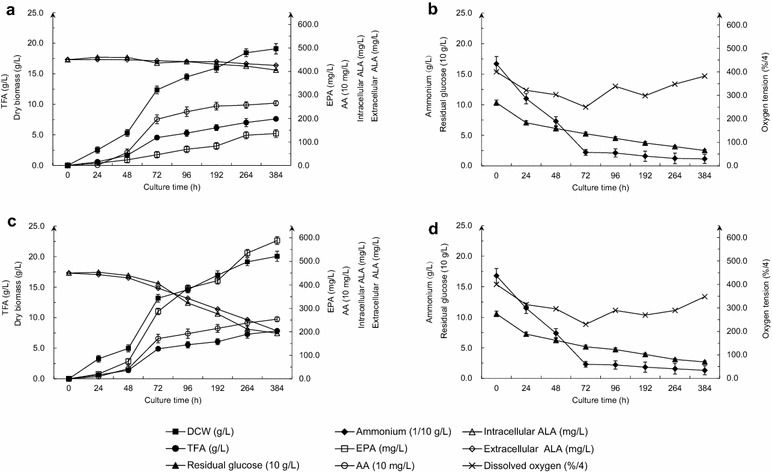
Time course of DCW, TFA, EPA level, AA level, intracellular ALA and extracellular ALA in submerged fermentation with WT *M. alpina* (**a**) and Ma-MpFADS6-2 (**c**), and residual glucose, ammonium and dissolved oxygen (DO) in WT *M. alpina* (**b**) and Ma-MpFADS6-2 (**d**) at 28 °C for 3 days and then at 12 °C for 8 days with an aeration rate of 0.5 vvm and agitation at 500 rpm, and finally aging at 12 °C for another 5 days. All values are the means of three determinations and *error bars* represent standard deviations. TFA (g/L) = M_TFA_ of 50 mg freeze-dried cells × M_CDW_ of 50 mL cultures/50 mg × 20

The Ma-MpFADS6-2 transformant which produced the highest EPA level among all Ma-MpFADS6 transformants was selected to be inoculated in 5 L jar fermenter containing 4 L of fermentation medium; as a control, WT *M. alpina* was inoculated in parallel in a separate fermenter. After pre-cultivating at 28 °C for 3 days, fermenting at 12 °C for 8 days and aging at 12 °C for 5 days, there was no significant difference in DCW, TFA and AA levels between the two samples in the whole fermentation (Fig. 6a, c), whereas there was a noticeable increase in DCW, TFA and AA levels in shaking flasks (with 50 g/L PSM supplementation) (Figs. 5, 6). As shown in Fig. 6b, the selected Ma-MpFADS6-2 transformant grew continuously from 0 to 384 h and reached a maximal DCW of 20.1 ± 0.7 g/L at 384 h. The level of intracellular ALA in WT *M. alpina* decreased slowly to 405.7 mg/L (Fig. 6a). By contrast, in the Ma-MpFADS6-2 transformant, it decreased as the fermentation went on, with a faster decline from 72 to 96 h (Fig. 6c). At the end of the fermentation, only 193.8 ± 26.6 mg/L intracellular ALA remained. The level of extracellular ALA in the Ma-MpFADS6-2 transformant was also decreased, and the remaining amount was similar to intracellular ALA. Moreover, the Ma-MpFADS6-2 transformant accumulated EPA slowly between 0 and 48 h, then the EPA yield increased after 48 h. The maximal EPA yield in the Ma-MpFADS6-2 transformant reached 588.5 ± 29.6 mg/L (30.2 mg/g, compared to 2.1 mg/g in WT *M. alpina*) at 384 h, which was 73 mg/L higher than the yield obtained in shaking flasks supplemented with 50 g/L PSM (Figs. 5c, 6c). The EPA yield of WT *M. alpina* showed a roughly linear increase, and the maximal EPA reached was only 135.6 ± 16.9 mg/L at the end of fermentation (Fig. 6a). EPA productivity was enhanced to 36.72 mg/L/d in the Ma-MpFADS6-2 transformant culture, which was 4.3-fold higher than that in WT *M. alpina* culture (8.40 mg/L/d). The Ma-MpFADS6-2 transformant and WT *M. alpina* produced 11.8 and 2.7 mg EPA per g of PSM, respectively.

As the fermentation continued, nitrogen nutrients were consumed rapidly within 72 h, then nitrogen consumption slowed down, and only a low amount remained after 72 h. Carbon nutrients were consumed linearly during cell growth and PUFAs production; the amount of glucose remaining at the end of fermentation was 12.6 ± 1.5 g/L in WT *M. alpina* and 13.5 ± 1.6 g/L in Ma-MpFADS6-2 cultures (Fig. 6b, d). During the first 72 h of fermentation, the level of dissolved oxygen decreased to about 60 %. When the fermentation temperature was switched to 12 °C, dissolved oxygen decreased once more following a significantly increased between 72 and 96 h, and increased to about 90 % at the end of the fermentation (Fig. 6b, d).

## Discussion

Very long chain (VLC)-PUFA synthesis in *M. alpina* proceeds predominantly through the FADS6 pathway, in which the first committed step is the delta-6 desaturation (Additional file 1: Fig. S1), and FADS6 is generally considered to be a rate-limiting enzyme for this pathway [15, 16]. The substrate preference of FADS6 determines whether LA can be further converted to ω6-PUFAs or ALA to ω3-PUFAs. Our previous study showed that MaFADS6-I had substrate preference for LA and not for ALA [11]. Therefore, we reasoned that transformation of an exogenous ALA-preferring gene into *M. alpina* may be the key to the accumulation of higher ω3-PUFA levels. However, due to the low ALA level in vivo (which is probably the reason why delta-15 desaturase in *M. alpina* has a low activity for LA), our result revealed that the EPA level was not readily increased in substrate-free cultures, and exogenous ALA had to be supplemented as the substrate for the ALA-preferring FADS6. Linseeds, perilla seeds, sesame seeds and peony seeds are rich in ALA, and among these, peony seeds have been reported to contain the highest percentage of ALA, where it reaches more than 40 % of TFA [17]. Tree peony has been widely planted over 20,267 hectares in China due to its superior ornamental characteristics, with a potential annual seed production of 57,855 tons [17]. However, tree peony seed oil (PSO) has seen little use so far, and the byproducts of oil processing have not been fully exploited. *Mortierella alpina* is capable of using a variety of substrates for its growth and EPA production, including linseed oil [18], crude soybean oil [13], vegetable oils [19], sunflower press cake [20], fish meal and soybean meal [21]. In our study, cultures of WT *M. alpina* and transformants were supplemented with no substrate, the free form of the ALA fatty acid, and different concentrations of PSO or PSM for enhancing EPA production (Additional file 5: Fig. S5).

In the absence of exogenous substrate, the level of intracellular ALA in *M. alpina* and all transformants was very low (Fig. 2d), while EPA production in Ma-MpFADS6 transformants was increased fourfold compared to WT *M. alpina*, from 22.5 ± 2.9 to 80.1 ± 3.2 (Fig. 2f), suggesting that ALA consumption catalyzed by MpFADS6 altered the reaction balance of intracellular fatty acids biosynthesis, further promoting the conversion of LA to ALA and increasing the level of intracellular ALA to be converted to more PUFAs. The reaction reached an equilibrium once more and EPA production was no longer increased and the residual amount of intracellular ALA was still low. Extracellular fatty acid was not detected, suggesting that *M. alpina* did not have the ability to secrete any form of lipid. EPA production in Ma-MpFADS6 transformants was a little higher at low temperature than at constant temperature (Fig. 2f), suggesting that the low temperature did not decrease the activity of MpFADS6. This is probably the reason why FADS6 isozymes play a physiologically important role in adaptation to low temperatures [22]. This result also suggested that the activity of MpFADS15 was slightly higher, perhaps due to its preference for C20 rather than C18.

After supplementing the free fatty acid form of ALA, EPA production in Ma-MpFADS6 transformants was significantly improved (Fig. 3d), whereas their remaining level of intracellular ALA was lower than in WT *M. alpina* (Fig. 3f), indicating that MpFADS6 can utilize exogenous free ALA as its substrate and convert it to SDA. However, the fatty acid profile in all strains showed only small amounts of intermediates (SDA and ETA), suggesting that the following delta-6 elongation and delta-5 desaturation in *M. alpina* were not rate-limiting steps and ALA was readily converted to EPA (Additional file 1: Fig. S1). In all the samples with ALA addition, the sum of intracellular ALA and EPA levels in WT *M. alpina* and Ma-MaFADS6-I transformants reached only about 50–75 % of the total amount of ALA added, and notably, less than half in WT *M. alpina* (Fig. 3d, f). Unexpectedly, little extracellular ALA was detected in the supernatant. It was suspected that this low level resulted from the self-oxidation of the fatty acid substrate in culture. To examine this hypothesis, the free form of LA or ALA was added to sterilized medium alone for an 8-day mock cultivation. The result showed that ninety percent of LA and fifty percent of ALA remained after shaking under the same conditions (data not shown), suggesting that both substrates were partially oxidized over the culture time.

Considering the high cost and the propensity of free fatty acids to undergo self-oxidation, PSO was supplemented in triacylglycerol (TAG) form as an exogenous substrate. After supplementing media with lower concentrations of PSO in Ma-MpFADS6 transformants, EPA yield was significantly improved, indicating that *M. alpina* can use TAG as a substrate. However, after supplementing cultures with higher concentration of PSO, not only was EPA production lower than with lower concentrations of PSO, but unexpectedly the level of AA was sharply reduced to less one-fourth of that with lower concentrations of PSO (Fig. 4c, d). This may be due to a toxic effect of high PSO concentrations on the lipid production in *M. alpina*, but the fungal biomass was not affected. The level of intracellular ALA in Ma-MpFADS6 transformants was equivalent to that in *M. alpina* (Fig. 4e), suggesting that a high concentration of PSO inhibited the activity of MpFADS6. The sum of intra- and extracellular ALA levels was nearly equal to the total amount of ALA added, suggesting that the TAG form of ALA was not oxidized, and the PSO in glycerolipid form was much more stable in culture than free ALA.

PSM addition was another key to improvements in EPA production. After supplementing cultures with PSM, the DCW was significantly increased, since PSM contains a large amount of carbon and nitrogen nutrients. PSM is a by-product of PSO extraction and presents some residual ALA-containing oil that can be used as the precursor for EPA synthesis in transgenic *M. alpina*. Thus, EPA production was increased Ma-MpFADS6 transformants (Fig. 5d), and the percentage of EPA was raised slightly relative to WT *M. alpina* (Fig. 5f), suggesting that the increase in EPA production was mainly the result of a higher DCW. The decrease in extracellular ALA level during fermentation illustrates the fact that extracellular ALA continued to enter the cells as intracellular ALA was being consumed.

In the transformation of an exogenous gene into *M. alpina*, the selection of a suitable transformation method and construction of the *M. alpina* genetic manipulation system are also key for EPA accumulation. There are many transformation methods available for filamentous fungi [23, 24], including protoplasts/PEG, lithium acetate, electroporation, biolistics, restriction enzyme mediated integration (REMI) and ATMT. Recently, many types of *M. alpina* genetic manipulation system have been constructed to enhance the transformation efficiency and overexpression of exogenous genes [25, 26]. *M. alpina* uracil-auxotrophic strain was isolated and binary plasmid pBIG2-ura5s-ITs was successfully constructed for ATMT in our laboratory, and the malic enzyme gene of *M. alpina* was overexpressed using this genetic expression system [27]. However, our *M. alpina* genetic manipulation system can transform a single gene into host strain by far. We are realizing multigene transformation in *M. alpina* uracil-auxotrophic strain, thereby engineering the metabolic pathway of *M. alpina* for the EPA production in substantial quantities. Exogenous ω3 desaturase, highly active at constant temperature and preferring 18-C substrates, will be transformed into *M. alpina* to work together with MpFADS6, and knockout of endogenous MaFADS6 will block the ω6 pathway to reduce LA consumption for AA production, so that LA can only be converted to ALA and then EPA can be accumulated by MpFADS6. We anticipate that transformation of an exogenous ω3 desaturase and knockout of endogenous MaFADS6 can further increase EPA production in *M. alpina*.

Ando et al. [28] over-expressed ω3-desaturase gene in *M. alpina* 1S-4 by employing the ATMT system, and EPA reached a maximum of 40 % of total fatty acids (0.68 g/L) when transformants were cultivated in 4 mL GY medium for 16 days. Recently, Tomoyo et al. [29] transformed Δ17 desaturase of *Saprolegnia diclina* into *M. alpina*, which was inoculated in a 20 mL flask containing 4 mL GY medium and yielded 1.8 g/L of EPA under low temperature conditions. But most of the liquid in the culture had been evaporated after 7 days cultivation in our preliminary experiments. The EPA yields of our experiments were based on the larger flasks and 5 L fermenter, and we could not obtain such results from the published data.

Compared to their works, our study is the first report demonstrating the accumulation of EPA in *M. alpina* via manipulation of the ω3 pathway. Although only 588.5 ± 29.6 mg/L EPA was achieved in *M. alpina* ATCC 32222, there is a potential to significantly lower the overall cultivation costs for EPA production by addition of inexpensive PSM. One gram PSM, as a waste by-product, was converted to a high value-added commodity containing 11.8 mg EPA by microbial fermentation. This high value-added PSM can be better used in animal feed. The AA level of all transformants was not altered when EPA was produced, and our result showed that the EPA/AA ratio ranged from 1:20 in *M. alpina* to 1:4 in the Ma-MpFADS6-2 transformant, which enhanced the ω-3/ω-6 ratio for supplement applications and would be reasonable for the optimal ω3/ω6 ratio at ≈1:4 [30].

## Conclusions

In this study, we have successfully overexpressed the MpFADS6 gene in *M. alpina* uracil-auxotrophic strain, representing the first report of EPA bioproduction via the ω3 pathway of *M. alpina.* EPA production in Ma-MpFADS6 transformants was enhanced to various degrees by supplementing cultures with no substrate, free form of fatty acid, different concentrations of PSO or PSM (Additional file 6: Fig. S6). Conversion of byproduct (PSM) from the PSO industry to high-value lipid by microbial fermentation proves to be an economically viable option for EPA production.

## Methods

### Strains and plasmids

Wild-type (WT) *M. alpina* (ATCC32222; DDBJ/EMBL/GenBank accession no. ADAG00000000 [first version]) was purchased from ATCC (American type culture collection) and conserved in our lab; *M. alpina* uracil-auxotrophic strain (CCFM501) was modified from *M. alpina* by Guangfei [27] in our lab. MpFADS6 gene (GenBank accession KU236373) was synthesized by Shanghai Sunny Biotechnology Co. Ltd; *Agrobacterium tumefaciens* C58C1 (*A. tumefaciens*), which was kindly provided by Yasuyuki Kubo (Kyoto Prefectural University, Japan), was used as a transfer DNA (T-DNA) donor for fungal transformation; pBIG2-ura5s-ITs plasmid was modified in our lab from pBIG2RHPH2 sent from Yasuyuki Kubo.

### Media and cultural conditions

WT *M. alpina* was grown on potato dextrose agar (PDA) medium at 28 °C. *Mortierella alpina* uracil-auxotrophic strain was maintained on GY medium, consisting of 30 g/L glucose, 5 g/L yeast extract, 2 g/L KNO_3_, 1 g/L NaH_2_PO_4_ and 0.3 g/L MgSO_4_·7H_2_O, supplemented with 0.05 c uracil. YEP medium, consisting of 10 g/L tryptone, 10 g/L yeast extract, and 5 g/L NaCl, was used for *A. tumefaciens* cultivation at 28 °C. Synthetic complete (SC) medium was used for screening positive transformants. Minimal medium (MM) and induction medium (IM) were used for co-cultivation of *M. alpina* and *A. tumefaciens*. The composition of these media were described previously [31]. For the fatty acid analysis, each transformant and *M. alpina* were grown for 7 days at 28 °C in Broth medium, consisting of 20 g/L glucose, 5 g/L yeast extract, 1 g/L KH_2_PO_4_, 0.25 g/L MgSO_4_·7H_2_O, 10 g/L KNO_3_, pH 6.0. Lipid producing medium, consisting of 50 g/L glucose, 13.3 g/L KNO_3_, 3 g/L KH_2_PO_4_, 1 g/L Na_2_SO_4_, 0.5 g/L MgCl_2_·6H_2_O, 0.5 g/L CaCl_2_·2H_2_O, pH 6.0, was used for oil-producing cultivation. PSO (0.025, 0.05, 0.1 or 0.2 %, v/v) with 0.05 % Tween 80 or PSM (12.5, 25 or 50 g/L) were added in lipid producing medium as exogenous substrates for lipid production. 50 g/L PSM was added in lipid producing medium for jar fermentation (fermentation medium). PSM in medium mentioned above was prepared by the following procedure: PSM was crushed using a mortar/pestle, sieved through a 40 mesh, boiled in double distilled water, precipitated for 10 min and filtered through quadruple layers of gauze.

### T-DNA binary plasmid construction, genetic transformation by ATMT and transformant screening

MaFADS6-I and MpFADS6 genes were amplified with pBIG-FMa/pBIG-RMa primers and pBIG-FMp/pBIG-RMp primers, respectively, as shown in Table 1. The PCR was carried out in a total volume of 50 μL. After initial denaturation at 94 °C for 4 min, amplification was performed in 30 cycles of 40 s at 94 °C, 40 s at 55 °C, and 2 min at 68 °C, followed by a final extension at 68 °C for another 5 min. Amplification products were fractionated on 1.0 % agarose gels and subcloned into the vector pBIG2-ura5s-IT1 between the TrpCter promoter and the trpCt terminator for overexpression in *M. alpina* uracil-auxotrophic strain. MaFADS6-I was digested and subcloned into *Hin*dIII and *Xho*I sites to generate a plasmid designated pBIG2-ura5s-MaFADS6-I while MpFADS6 was subcloned into *Hin*dIII and *Sma*I sites to generate a plasmid designated pBIG2-ura5s-MpFADS6.

**Table 1:** Primers used in this study

Primer name	Restriction enzyme	Oligonucleotide sequence (5′–3′)^a^	Function
pBIG-FMa	*Hin*dIII	CGGCCGAAGCTTATGGCTGCTGCTCCCAGTGTGAGGAC	MaFADS6-I amplification for binary plasmid construction
pBIG-RMa	*Xho*I	CGGGGCCTCGAGTTACTGCGCCTTACCCATCTTGGAGG	
pBIG-FMp	*Hin*dIII	TACCGAAGCTTATGTGCCCGCCGAAGACGGACGGC	MpFADS6 amplification for binary plasmid construction
pBIG-RMp	*Stu*I	TAAAAGGCCTTCAGTGCGCCTTCTCCGCCTTGCCG	
FMa	–	ATGGCTGCTGCTCCCAGTGTGAGGAC	Target genes insert detection for binary plasmid construction
RMa	–	TTACTGCGCCTTACCCATCTTGGAGG	
FMp	–	ATGTGCCCGCCGAAGACGGACGGC	
RMp	–	TCAGTGCGCCTTCTCCGCCTTGCCG	
HisproF	–	CACACACAAACCTCTCTCCCACT	T-DNA insert detection for binary plasmid construction
TrpCR	–	CAAATGAACGTATCTTATCGAGATCC	
RT-MaD6-F	–	ATGGCTGCTGCTCCCAGTGTGAGGAC	qPCR for MaFADS6-I transcript level measurement
RT-MaD6-R	–	CACCTTGTTGTCGATGATCATCAGGAAGGG	
RT-MpD6-F	–	ATGTGCCCGCCGAAGACGGAC	qPCR for MpFADS6 transcript level measurement
RT-MpD6-R	–	CGTTTTGAGATCGACGGGCGCG	
18SRTF	–	CGTACTACCGATTGAATGGCTTAG	Internal control gene for qPCR
18SRTR	–	CCTACGGAAACCTTGTTACGACT	

^a^Underlined sequences indicate additional restriction sites

The binary plasmids (pBIG2-ura5s-MaFADS6-I and pBIG2-ura5s-MpFADS6) were transferred into *A. tumefaciens* C58C1 strain by electrotransformation. The detailed procedures of the ATMT method is described in Michielse et al. [31]. Co-cultivation of *A. tumefaciens* harboring pBIG2-ura5s-MaFADS6-I or pBIG2-ura5s-MpFADS6 and *M. alpina* uracil-auxotrophic strain, genomic DNA preparation and confirmation of the existence of T-DNA in the genome of transformants were performed following our previously published methods [27].

### RT-qPCR analysis for relative transcript level of transformants

Total RNA of transformants and WT *M. alpina* was extracted using the *HYQspin*™ Total RNA Kit (HG403-03) and reverse-transcribed using the QuantScript RT kit (TIANGEN) according to the manufacturer’s instructions. Primers used for RT-qPCR are shown in Table 1. RT-qPCR was performed on an ABI-Prism 7900 sequence detection system with Sup-qPCR Master Mix (SYBR Green, QR01C) according to the manufacturers’ instructions. The PCR amplification involved 50 °C for 2 min, 95 °C for 10 min, followed by 40 cycles of 95 °C for 15 s and 60 °C for 30 s. The expression of the 18S rDNA gene was used as the internal control (seen in Table 1). The 20-μL reaction mixture consisted of 10 μL of SYBR Green PCR Master Mix, 0.5 μL of each primer pairs, 1 μL of DNA template or distilled water as a negative control and 8.5 μL of distilled water. The transcript levels were calculated using the 2^−ΔΔCt^ method [32].

### Culture condition of transformants for PUFAs production in the presence of exogenous substrates

Each stable transformant and WT *M. alpina* were cultivated in 50 mL Broth medium at a shaking speed of 200 rpm at 28 °C for 7 days (constant temperature) or at 28 °C for 3 days and then at 12 °C for 8 days (low temperature). The free form of LA or ALA was added in Broth medium as a substrate with a final concentration of 0.1 mg/mL for free fatty acid investigations. PSO (0.025, 0.05, 0.1 or 0.2 %, v/v) with 0.05 % Tween 80 or PSM (12.5, 25 or 50 g/L) were added in lipid-producing medium as exogenous substrates for triglyceride investigations. All strains were grown at shaking speed of 200 rpm at 28 °C for 3 days and then at 12 °C for 8 days (low temperature). All experiments were carried out in triplicate.

### Bench scale production of EPA

One of the three Ma-MpFADS6 transformants with the highest EPA production and WT *M.alpina* (control) were grown in lipid-producing medium with 50 g/L PSM in a 5-L jar fermenter containing 4 L of fermentation medium (50 g/L glucose, 50 g/L PSM, 13.3 g/L KNO_3_, 3 g/L KH_2_PO_4_, 1 g/L Na_2_SO_4_, 0.5 g/L MgCl_2_·6H_2_O, 0.5 g/L CaCl_2_·2H_2_O, pH 6.0) at 28 °C for 3 days and then at 12 °C for 8 days with an aeration rate of 0.5 vol/vol/min (vvm) and agitation at 500 rpm, and finally aged at 12 °C for another 5 days. Cells of the Ma-MpFADS6 transformant and WT *M. alpina* were collected at 0, 24, 48, 72, 96, 120, 192, 264 and 384 h for determination of the growth curve, level of total fatty acids (TFA), AA, ALA and EPA, residual glucose, ammonium and dissolved oxygen (DO).

### Lipid extraction and fatty acid analysis

For the extraction of fatty acids, cells were centrifuged at 10,000×*g* and lyophilized. Lipids from an equivalent weight of freeze-dried cells (50 mg) and supernatant were extracted and methyl esterified as described previously [33]. Fatty acid methyl esters (FAMEs) were analyzed by GC. Pentadecanoic acid (C15:0, NU-CHEK) and Heneicosanoic acid (C21:0, NU-CHEK) were added to the biomass samples as the internal standard to quantify the fatty acid content. GC analysis was performed with a GC-2010 (Shimadzu Co. Japan) equipped with a FID detector and a capillary DBWAX column (30 m × 0.32 mm, φ 0.25 μm; Agilent, USA). The samples were measured with a split of 20:1 with the injector temperature set to 240 °C. The column temperature was 180 °C. FAMEs were identified by comparing with commercial FAME standards (37-component FAME Mix, Supelco, USA). Fatty acid profiles of PSO and PSM were determined by the same method as above.

## Additional files

10.1186/s12934-016-0516-5 Aerobic VLC (very long chain) PUFAs biosynthetic pathways modified from Napier [34] including microorganisms, plants and animals. The various routes for biosynthesis of AA, EPA, and DHA are shown, as mediated by the consecutive action of desaturases and elongases. The predominant delta 6-pathway is shown in black boxes with bold white font, as is the alternative D8-pathway. ALA-rich PSO or PSM was added as exogenous substrate in medium to supply a sufficient source of ALA. Three routes for DHA biosynthesis are shown: microbial D4 pathway, mammalian “Sprecher” pathway and polyketide synthase (PKS) pathway (PUFAs are synthesized by polyketide synthase controlling chain length). There is no alternative delta 8 pathway or microbial delta 4 pathway in *M. alpina*. ELOVL = elongation of long-chain fatty acids, FADS9 = delta 9 desaturase, ELO9 = delta 9 elongase, FADS12 = delta 12 desaturase, FADS6 = delta 6 desaturase, FADS15 = delta 15 desaturase, ELO6 = delta 6 elongase, FADS8 = delta 8 desaturase, FADS5 = delta 5 desaturase, FADS17 = delta 17 desaturase, ELO5 = delta 5 elongase, FADS19 = delta 19 desaturase, FADS4 = delta 4 desaturase, ELO7 = delta 7 elongase.
10.1186/s12934-016-0516-5 Construction of binary vectors pBIG2-ura5s-MaFADS6-I (c) and pBIG2-ura5s-MpFADS6 (d). The his 550 promoter fragment was derived from *M. alpina* ATCC 32222. The trpCt transcription terminator region was derived from *Aspergillus nidulans*. LB, left border; RB, right border. The binary vector pBIG2-ura5s-IT1 was constructed by Guangfei in our lab [27]. (a) and (b): Cloning of MaFADS6-I and MpFADS6. M: Marker; 1, 2: MaFADS6-I; 3, 4: MpFADS6. (e) and (f): Confirmation of binary vectors pBIG2-ura5s-MaFADS6-I and pBIG2-ura5s-MpFADS6 using MaFADS6-I and MpFADS6 primers. M: Marker; 5, 6: MaFADS6-I; 7: positive control (MaFADS6-I); 8, 9, 10: MpFADS6; 11: positive control (MpFADS6). (g) and (h): Confirmation of binary vectors pBIG2-ura5s-MaFADS6-I and pBIG2-ura5s-MpFADS6 using HisproF/TrpCR primers. M: Marker; 12, 15: negative control (pBIG2-ura5s-ITs); 13, 14: pBIG2-ura5s-MaFADS6-I; 16, 17, 18: pBIG2-ura5s-MpFADS6.
10.1186/s12934-016-0516-5 The fatty acid profiles (expressed as percentage) in PSO and PSM extracts. PSO contains 46.65 % ALA, 26.85 % LA, 19.78 % OA, 5.08 % PA and 1.25 % SA, and the TFA level (consisting of 38.88 % ALA, 23.14 % LA, 20.49 % OA, 5.45 % PA and 1.43 % SA) accounts for 4 % of the total PSM mass.
10.1186/s12934-016-0516-5 Extracellular ALA level of transformant and WT *M. alpina* cultures in flasks with different concentrations of PSO (a) and PSM (b) at low temperature.
10.1186/s12934-016-0516-5 Gas chromatogram of fatty acids in control and recombinant cells. WT *M. alpina* (a) and Ma-MpFADS6 transformant (b) with free substrate under low temperature conditions; WT *M. alpina* (c) and Ma-MpFADS6 transformant (d) with free ALA; WT *M. alpina* (e) and Ma-MpFADS6 transformant (f) with 0.1 % PSO; WT *M. alpina* (g) and Ma-MpFADS6 transformant (h) with 50 g/L PSM.
10.1186/s12934-016-0516-5 The summarized graph for DCW (a), TFA (b), EPA level (c) and the percentage of EPA (d) in *M. alpina* and Ma-MpFADS6 transformants with no substrate in constant temperature and low temperature, free form of fatty acid, PSO and different concentrations of PSM. Data and error bars of Ma-MpFADS6 represented the average and standard deviation for Ma-MpFADS6-1/2/3, respectively.

## Abbreviations

PUFAs: polyunsaturated fatty acids
FADS6: delta-6 desaturase
LA: linoleic acid (18:2^Δ9,12^)
AA: arachidonic acid (20:4^Δ5,8,11,14^)
ALA: α-linolenic acid (18:3^Δ9,12,15^)
EPA: eicosapentaenoic acid (20:5^Δ5,8,11,14,17^)
DHA: docosahexaenoic acid (22:5^Δ4,7,10,13,16,19^)
GLA: γ-linolenic acid (18:3^Δ6,9,12^)
SDA: stearidonic acid (18:4^Δ6,9,12,15^)
DGLA: dihomo-γ- linolenic acid, (20:3^Δ8,11,14^)
*M. alpina*: *Mortierella alpina* ATCC32222
